# Draft Genome Sequence of *Bacillus obstructivus* VT-16-70 Isolated from the Bronchoalveolar Lavage Fluid of a Patient with Chronic Obstructive Pulmonary Disease

**DOI:** 10.1128/genomeA.01754-16

**Published:** 2017-03-02

**Authors:** Victor Tetz, George Tetz

**Affiliations:** Human Microbiology Institute, New York, New York, USA

## Abstract

We report here the draft genome sequence of *Bacillus obstructivus* VT-16-70, a novel spore-forming bacterium isolated from the lungs of a patient with chronic obstructive pulmonary disease. The genome comprised 5,220,753 bp, with 35.2% G+C content. There were 4,972 predicted protein-coding genes, including those associated with antibiotic resistance and virulence.

## GENOME ANNOUNCEMENT

*Bacillus* is a genus of spore-forming, motile, Gram-positive, aerobic, and rod-shaped bacteria. *Bacillus* species are associated with a number of human pathologies, such as *Bacillus anthracis*, which is the causative agent of anthrax, and *Bacillus cereus*, which is now recognized as a cause of endocarditis, meningitis, and other diseases (1–3). However, members of the *Bacillus* family have been poorly explored (4–6).

The 16S rRNA gene of *Bacillus obstructivus* VT-16-70, which was isolated from the bronchoalveolar lavage fluid from a patient with chronic obstructive pulmonary disease, was sequenced and found to possess 98% sequence identity with that of *Bacillus acidicola*. However, matrix-assisted laser desorption ionization–time of flight mass spectrometry (MALDI-TOF MS) analysis indicated low similarity of the *B. obstructivus* 16S rRNA gene with that of *Bacillus oleronius*. The whole genome of *B. obstructivus* VT-16-70 was sequenced by using Illumina HiSeq 2500 sequencing technology (Illumina GA IIx; Illumina, CA). Library preparation, sequencing reactions, and runs were carried out according to the manufacturer’s instructions. A draft genome was assembled by using SPAdes version 3.5.0, with 114-fold average coverage (7).

The assembled 448 contigs totaled 5,220,753 bp, with a G+C content of 35.2%. The assembled sequences were annotated using the NCBI Prokaryotic Genome Annotation Pipeline and Rapid Annotations using Subsystems Technology (RAST) (7, 8). The genome harbored 162 tRNA genes, 17 rRNA and five noncoding RNA (ncRNA) operons, and 4,972 protein-coding sequences. The analysis revealed the presence of multidrug resistance transporters of the ABC, multidrug and toxic compound extrusion (MATE), and major facilitator superfamily (MFS) families, and genes conferring resistance to antibiotics, including aminoglycosides, daunorubicin, fosfomycin, oxetanocin, beta-lactam antibiotics, tetracycline, and hydroperoxides. Virulence factors, including hemolysin D, proteases, peptidases, deoxyribonucleases, ribonucleases, and adhesins, in addition to capsular, flagellar, and sporulation proteins (9–11), were identified in the genome.

In comparison with the genome of the closest relative, *B. acidicola*, the genome of* B. obstructivus* VT-16-70 is smaller (5,138,363 bp versus 5,220,753 bp) and has significantly higher G+C content (39.4% versus 35.2%). An* in silico* DNA-DNA hybridization (DDH) analysis confirmed that the genomes of *B. obstructivus* VT-16-70 and *B. acidicola* belonged to two different species, as the Genome-to-Genome Distance Calculator algorithm produced a DDH value of 25.40%, which is well below the threshold value of 70% (12, 13).

Further studies of *B. obstructivus* VT-16-70 will result in a better understanding of its role in the microbiome of chronic obstructive pulmonary disease, as well as the possible pathogenicity of this bacterium.

### Accession number(s).

The complete genome sequence has been deposited in the NCBI database under accession no. MPHG00000000.
